# Economic Evaluation of Acute Appendicitis Therapeutic Interventions: A Systematic Review

**DOI:** 10.1002/hsr2.70815

**Published:** 2025-05-05

**Authors:** Hasan Abolghasem Gorji, Sajad Moeini, Mohmmad Veysi Sheikhrobat, Aziz Rezapour, Aghdas Souresrafil, Mohammad Barzegar

**Affiliations:** ^1^ Department of Health Services Management School of Health Management and Information Sciences, Iran University of Medical Sciences Tehran Iran; ^2^ Health Management and Economics Research Center, Health Management Research Institute Iran University of Medical Sciences Tehran Iran; ^3^ Department of Health Services and Health Promotion, School of Health, Occupational Environment Research Center Rafsanjan University of Medical Sciences Rafsanjan Iran; ^4^ Department of English Language School of Health Management and Information Sciences, Iran University of Medical Sciences Tehran Iran

**Keywords:** acute appendicitis, cost‐effectiveness, economic evaluation, laparoscopic appendectomy, open appendectomy

## Abstract

**Background and Aims:**

Acute appendicitis (AA) is a prevalent cause of lower abdominal pain, often leading patients to seek emergency department care, particularly among young individuals. The present study aimed to systematically review cost‐effectiveness studies focusing on therapeutic interventions for AA.

**Method:**

Following the Preferred Reporting Items for Systematic Reviews and Meta‐Analyses (PRISMA) guidelines, we systematically reviewed economic evaluations of AA treatments published between 2000 and 2020. We searched multiple databases, including Cochrane, PubMed, Scopus, and Web of Science. The studies included in this review were assessed using the Quality of Health Economic Studies (QHES) checklist, and cost data were standardized to 2022 US dollars.

**Results:**

Out of the 53 screened studies, 11 fulfilled the inclusion criteria. The studies’ average QHES score was of high quality (0.87). Most studies were from the payer's perspective and the health system (four studies each). Five studies were based on the decision tree model, and three were based on the Markov model. Four studies were conducted on children. Of the 11 studies reviewed, five support the cost‐effectiveness of laparoscopy, five support the cost‐effectiveness of antibiotic therapy, and one supports the cost‐effectiveness of open appendectomy.

**Conclusions:**

Based on the findings of this study, laparoscopic therapeutic intervention, compared to open appendectomy, can be more cost‐effective for the treatment of patients with AA.

## 1 Introduction

Acute appendicitis (AA), which is one of the leading causes of lower abdominal pain, causes patients to go to the emergency department, and it is the most commonly diagnosed disease among young patients who are hospitalized with acute abdominal pain [1]. Approximately 7%–10% of the general population experiences AA, with the highest incidence occurring in the second and third decades of life, and 7%–10% of all emergency department visits are due to acute abdominal pain [2, 3]. Different geographical areas are reported to have different infection risks. United States residents are reported to be at an 8% lifetime infection risk, while European and African residents are at a 2% lifetime infection risk [4]. There are also differences among countries in per capita income, disease severity, and types of surgical management for affected patients [5].

Historically, open appendectomy was used to treat AA; however, laparoscopic appendectomy has become the surgical standard of care for 15–20 years in most middle‐ and high‐income countries [6]. Additionally, nonoperative management with antibiotics has emerged as a potential alternative, particularly in uncomplicated cases of AA [7]. By analyzing the complications and costs of surgical and nonsurgical interventions, Choosing the most favorable method remains challenging [8, 9].

The rising cost of healthcare has necessitated a focus on cost‐effectiveness in clinical decision‐making. Economic evaluations of treatment options for AA are essential for guiding healthcare policy and optimizing resource allocation. Although numerous studies have investigated the cost‐effectiveness of various treatment modalities, the findings have been inconsistent, reflecting differences in study design, cost components, and healthcare settings [10, 11, 12, 13, 14]. Given the global burden of AA and the critical need for cost‐efficient healthcare solutions, this systematic review aims to synthesize the available evidence on the economic evaluation of treatments for AA.

## 2 Methods

This systematic review followed the Preferred Reporting Items for Systematic Reviews and Meta‐Analyses (PRISMA) guidelines. The protocol for this review was registered with PROSPERO (CRD42023390140) to ensure transparency and adherence to standardized methodologies.

We systematically searched the Cochrane, PubMed, Scopus, Web of Science, CEA Registry databases, and Google Scholar to identify studies evaluating the economic aspects of treatments for AA. To enhance the comprehensiveness of our search, we also reviewed relevant organizational reports. The search was restricted to studies published between 2000 and 2022, reflecting the widespread adoption of laparoscopic appendectomy. A complete description of the search strategy is provided in Supporting Information S1: Appendix S1. After duplicate removal, two researchers independently screened the remaining studies’ titles, abstracts, and full texts. A third reviewer was consulted to resolve any disagreements.

The PICO framework was utilized to structure the systematic review question as follows: (P) Population: patients with AA; (I) Intervention: Open appendectomy; (C) Comparison: Laparoscopic appendectomy or nonoperative management; (O) Outcome: Cost per cost‐effectiveness unit, including Quality‐Adjusted Life Years (QALY) or Disability‐Adjusted Life Years (DALY).

### 2.1 Eligibility Criteria

In the present study, we included and reviewed only original studies and complete economic evaluation research (cost‐effectiveness analysis [CEA], cost‐benefit analysis [CBA], and cost‐utility analysis [CUA], and we excluded reviews, editorials, or protocols. Thus, we took account of economic evaluation studies assessing AA treatments (i.e., laparoscopic, nonoperative management, and open appendectomy) by model‐based and empirical economic evaluations.

### 2.2 Data Extraction

The same reviewers performed data extraction and analysis. For data extraction and categorization, we obtained the following pieces of information [1]: author name [2], publication year [3], country [4], cost per year of life gained (YLG) or QALY [5], study population [6], time horizon [7], perspective (e.g., healthcare system, healthcare provider, social, or payer) [8], discount rate [9], types of sensitivity analysis conducted [10], type of analysis [11], type of the model used [12], source of funding, and [13] willingness‐to‐pay (WTP) threshold per YLG or QALY.

Moreover, we evaluated all costs in US dollars (US$) to compare studies and cost‐effectiveness ratios. In addition, we converted The incremental cost‐effectiveness ratio (ICERs) and costs to US$2022 via the Campbell and Cochrane Economics Methods Group (CCEMG) [15].

### 2.3 Quality Assessment

Economic evaluation studies were evaluated based on the Quality of Health Economic Studies (QHES) checklist [16]. Two of the present researchers completed the QHES scale independently. Following the QHES checklist, they assigned a score of between 0 and 100. Four quality quartiles were allocated to the QHES scores: the 0–24 quartile (inferior quality), the 25–49 quartile (poor quality), the 50–74 quartile (moderate quality), and the 75–100 quartile (high quality) [16]. A third reviewer eliminated discrepancies with the questionnaire for data collection via discussions and all disagreements between the reviewers.

## 3 Results

**Figure 1:**
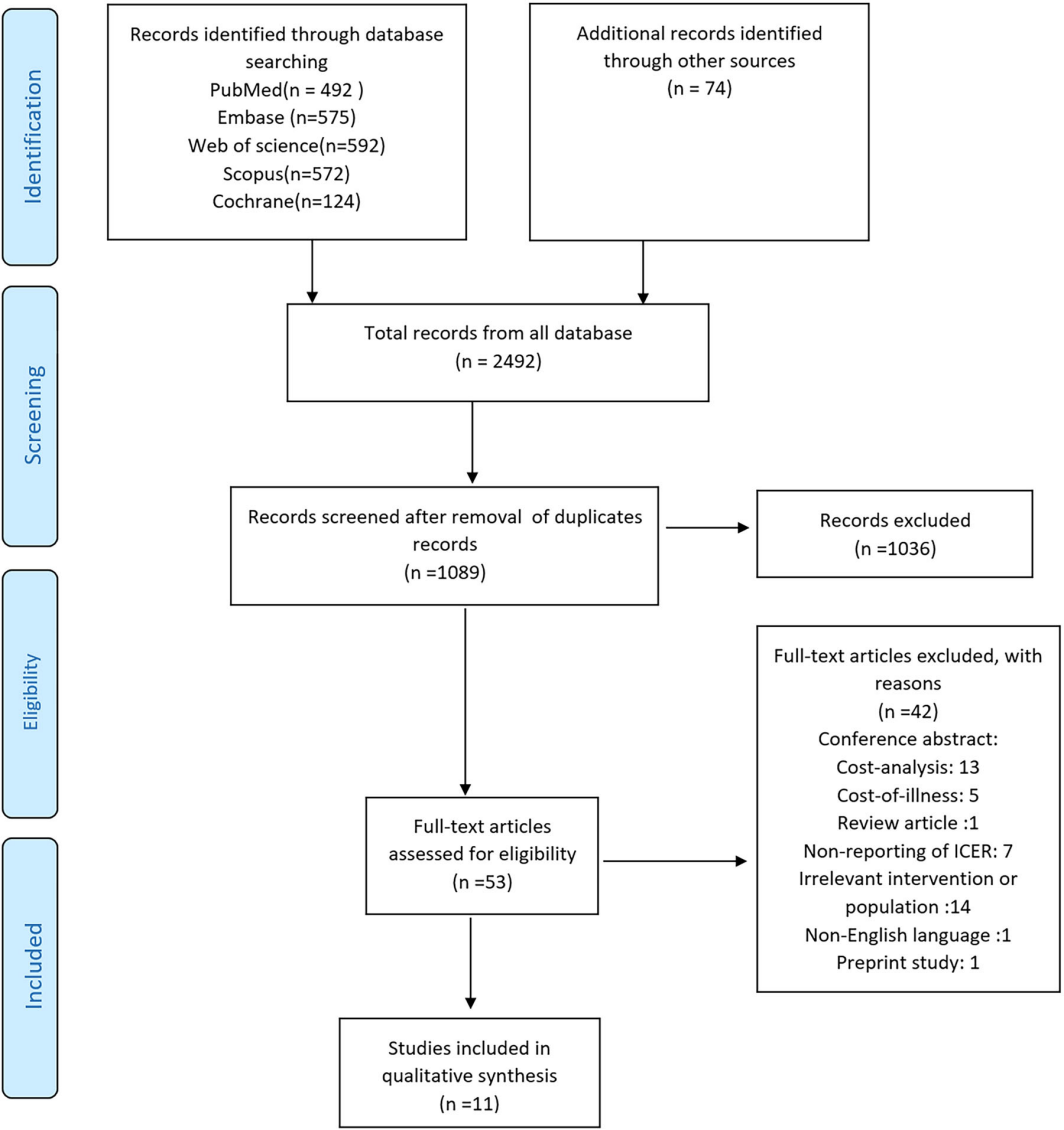
PRISMA flow chart for study selection.

In the initial stage of our review, we identified 2429 studies. After removing 1340 duplicates, we excluded 1036 studies based on title and abstract screening. A further 42 studies were excluded after a detailed examination of the full text. Ultimately, 11 studies met the inclusion criteria and were included in this systematic review, as shown in Figure 1 illustrates the study selection process.

The majority of the studies [6] were conducted in the USA [17, 18, 19, 20, 21, 22], with two studies carried out in Colombia [23, 24], and one study each in England [25], Japan [26], and Spain [27].

Regarding the perspective from which the studies were conducted, four studies were from the payers' perspective [18, 19, 23, 24], four from the health system perspective [20, 25, 26, 27], two from the service provider perspective [17, 22], and one from a social perspective [21].

In terms of the time horizon considered in the studies, six were conducted over 1 year [19, 20, 22, 23, 25, 26], with one study each spanning a lifetime [18], 10 years [27], 5 years [23], and 2 years [21].

Decision tree modeling was utilized in five studies [19, 20, 21, 22, 25], while Markov modeling was employed in three studies [18, 23, 26].

Probabilistic sensitivity analyses were conducted in seven studies simultaneously [18, 19, 20, 21, 23, 26, 27], and deterministic sensitivity analysis was performed in two studies [18, 23]. Eight CEAs [19, 20, 21, 22, 24, 25, 27] and three CUAs [22, 25, 27] were carried out.

The WTP thresholds varied across countries, with thresholds in the USA set at $50,000 and $100,000 per Quality‐Adjusted Life‐Year (QALY) [18, 19], US$107,000 in Japan [26], ₤20,000–₤30,000 in Spain, Colombia, and England [27], US$3000–US$6000 in Colombia [23, 24], and ₤23,000 per QALY in England [25].

The discount rates for costs and benefits reported in these studies ranged from 3% to 5%. Tables 1 and 2 provide detailed information on all the articles in this systematic review.

**Table 1:** Study design and setting overview.

Row	Study (year of publication)	Country	Comparators	Patient population	Costing year	Economic evaluation type	Time horizon	Perspective	Discount rate	Sensitivity analysis	Score of QHES
1	Ursula (2023)	US	Nonoperative management versus laparoscopic appendectomy in children	Children	2020	CEA (Decision tree)	1 year	Payer	NR	Yes, one‐way and PSA	0.91
2	Fuentes (2022) [27]	Spain	Laparoscopic versus open appendectomy	Children	NR	CUA	10 years	Health system	3	Yes, multivariate, and PSA	0.92
3	Guevara‑Cuellar (2021) [23]	Colombia	Nonoperative management versus open and laparoscopic surgery	Adults	2021	CEA (Markov model)	5 years	Payer	5	Yes, deterministic and PSA	0.93
4	Ali (2021) [25]	UK	Antibiotic therapy versus appendicectomy	Adults	2019	CUA (Decision tree)	1 year	Health system	NR	Yes, one‐way	0.87
5	Suzuki (2020)	Japan	Nonoperative management versus emergency laparoscopic appendectomy	Adults	2018	CEA (Markov model)	1 year	Health system	NR	Yes, one‐way and PSA	0.89
6	Sceats (2019) [18]	US	Operative versus nonoperative management	Adults	2017	CEA (Markov model)	Lifetime	Payer	3	Yes, one‐way deterministic and PSA	0.93
7	Alejandro (2017)	Colombia	Laparoscopic versus open appendectomy	Adults	2013	CEA	1 year	Payer	NR	NR	0.76
8	X. Wu (2017)	US	Nonoperative management versus laparoscopic	Children	2016	CEA (Decision tree)	2 years	Social	3	Yes, one‐way And PSA	0.93
9	Hartwich (2017)	US	Appendectomy versus nonoperative management	Adults	2015	CUA (Decision tree)	14 months	Healthcare	NR	NR	0.75
10	X. Wu (2015)	US	Nonoperative management versus laparoscopic	Children	2014	CEA (Markov model)	1 year	Health system	NR	Yes, one‐way and PSA	0.90
11	Haas (2012) [17]	US	Open versus laparoscopic	Adults	2005	CEA	NR	Healthcare	NR	Yes	0.75

Abbreviations: CEA, cost‐effectiveness analysis; CUA, cost‐utility analysis; NR, not reported; PSA, probabilistic sensitivity analysis.

**Table 2:** Findings of the cost‐effectiveness studies.

The first author (year of publication)	Comparators	Total cost (US$2022)	Mean of QALY/LYG	Main findings (US$2022)
Ursula (2023)	Nonoperative management	6803	0.997 QALY	ICER = $4,991,916/QALY (in support of antibiotic treatment)
	Laparoscopic	9666	0.996 QALY	
Fuentes (2022) [27]	Laparoscopic	2428 (Average cost)	0.77 QALY	ICER = $28,125/QALY results support laparoscopic treatment.
	Open appendectomy	1207 (Average cost)	0.75 QALY	
Guevara‑Cuellar (2021) [23]	Nonoperative Management	391	3.326 QALY	ICER: $25,527 (NM vs. LA) Laparoscopy has a lower cost than the other two methods and higher effectiveness than open appendectomy‐(in support of laparoscopy)‐
	Open appendectomy	370	3.331 QALY	
	Laparoscopic	400	3.333 QALY	
Ali (2021) [25]	Open appendectomy	3557	0.0424 QALY	In the option of antibiotic therapy instead of appendectomy, $35,724 is saved for each QALY obtained.
	Nonoperative Management	2508	0	
Suzuki (2020)	Nonoperative management	6831	0.9894 QALY	Nonoperative management without laparoscopy and with laparoscopy was not identified as cost‐effective compared to open (surgical) appendectomy.
	Open appendectomy	6576	0.984 QALY	
	Nonoperative management + laparoscopic	7641	0.9896 QALY	
Sceats (2019) [18]	Laparoscopic	8419	11.703 QALY 13.1668 LYG	Laparoscopy is recognized as a cost‐effective option over a lifetime horizon. Inpatient antibiotics cannot be an option in any way.
	Nonoperative management (outpatient)	187	11.665 QALY 13.1353 LYG	
	Nonoperative management (inpatient)	8157	11.666 QALY 13.1356 LYG	
Alejandro Ruiz‐Patino (2017)	Laparoscopic	78,006	271 Days (the number of days lost in a year)	ICER = $30 Incremental cost of $784 to treat 250 patients Incremental cost‐effectiveness equal to $30 in support of the cost‐effectiveness of laparoscopy
	Open appendectomy	77,217	297 Days (the number of days lost in a year)	
X. Wu (2017)	Nonoperative management	9974	23.53 QALY	ICER = $1,026,509/QALY nonoperative management is a more cost‐effective option than laparoscopy
	Laparoscopic	12,542	23.56 QALY	
Hartwich (2015)	Open appendectomy	4709	0.854 QALY	Incremental cost‐effectiveness between $50,175 and $222,369 per QALY saved (in support of antibiotic treatment)
	Nonoperative	3159	0.884 QALY	
X. Wu (2015)	Standard appendectomy	14,076	0.802 QALY	ICER of nonoperative with laparoscopy compared to routine surgery: $501,569/QALY
	Nonoperative management (with laparoscopic)	11,926	0.872 QALY	
	Nonoperative management (without Laparoscopic)	18,998	0.82 QALY	Cost of incremental effectiveness of nonoperative management without laparoscopy compared to routine management: less than $100,000 threshold.
				Generally, nonoperative management without laparoscopy is a cheaper and more effective option.
Haas (2012) [17]	Laparoscopic	12,295	—	Laparoscopy is $2536 less expensive than an appendectomy, while there is no significant difference in effectiveness.
	Open appendectomy	14,831	—	

Abbreviations: LYG, life years gained; QALY, quality‐adjusted life years.

### 3.1 Quality Assessment Findings

We employed the QHES checklist to evaluate the quality of the ultimate economic evaluation studies. Based on the obtained score, we also determined the studies' quality: 0–24: extremely low, 25–50: poor, 51–74: fair, 75–100: high. All the included studies received a score above 75, with a mean quality of 0.87 (SD = 0.12) (see Table 3), and they were, therefore, categorized as studies with high quality. Two studies did not mention their funding source [17, 22], and a few studies justified using the study perspective, a few studies justified using the study perspective, and seven studies did not report the discount rate, which is due to the 1‐year time horizon and nonapplicability of the discount rate [17, 19, 20, 22, 24, 25, 26].

**Table 3:** Quality assessment results of the included studies.

Study	Q1	Q2	Q3	Q4	Q5	Q6	Q7	Q8	Q9	Q10	Q11	Q12	Q13	Q14	Q15	Q16	Total score
Ursula (2023)	7	2	8	1	9	6	5	5	7	5	7	6	7	5	8	3	91
Fuentes (2022) [27]	7	2	8	1	9	6	5	7	8	5	7	6	6	5	7	3	92
Guevara‑Cuellar (2021) [23]	7	2	8	1	9	6	5	7	7	6	7	8	6	5	6	3	93
Ali (2021) [25]	7	2	8	1	9	6	5	5	7	5	7	7	5	5	5	3	87
Suzuki (2020)	7	2	8	1	9	6	5	5	7	5	7	6	6	5	7	3	89
Sceats (2019) [18]	7	2	8	1	9	6	5	7	8	6	7	7	6	5	6	3	93
Alejandro Ruiz‐Patino (2017)	7	2	7	1	5	6	5	5	6	5	6	5	5	4	6	3	76
X. Wu (2017)	7	2	8	1	9	6	5	7	7	5	6	8	7	5	7	3	93
Hartwich (2015)	7	2	8	1	6	6	4	5	7	5	6	6	5	5	6	0	75
X. Wu (2015)	7	2	8	1	9	6	5	5	7	5	6	8	7	5	6	3	90
Haas (2012) [17]	7	2	8	1	8	4	4	5	5	5	5	6	5	4	6	0	75

### 3.2 Cost‑Effectiveness Findings

Table 2 presents the cost‐effectiveness outcomes of treatments for AA based on four pediatric studies [19, 20, 21, 27]. The characteristics of these studies are summarized in Table 1. Three studies were conducted in the USA in 2015, 2017, and 2023. Adams et al. [19] examined the cost‐effectiveness of nonoperative management versus laparoscopic appendectomy in children with AA from the payer's perspective. Their decision tree model analysis revealed that nonoperative management was the dominant strategy in 97% of 10,000 Monte Carlo simulations, with a cost‐effective threshold of $100,000 per QALY compared to laparoscopy [19].

These three studies demonstrated that antibiotic therapy (nonsurgical disease management) was a more cost‐effective option for children. In Spain, Fuentes et al. [27] compared laparoscopic and open appendectomy in pediatric patients. They found that the average cost of treating AA with open appendectomy was €758, whereas for laparoscopy, it was €1525. The QALYs for appendectomy and laparoscopy were 0.75 and 0.77, respectively, resulting in an ICER of €18,000 per QALY in favor of laparoscopic treatment [27].

In Colombia, Guevara‐Cuellar et al. [23] reported that laparoscopy (363 ± 35 dollars) was less costly than open appendectomy (384 ± 41 dollars) and nonoperative management (392 ± 44 dollars). Nonoperative management (3.3310 QALY ± 0.057) was more effective than laparoscopy (3.3261 QALY ± 0.0707) and open appendectomy (3.3332 QALY ± 0.0276). Overall, laparoscopy emerged as the dominant strategy with the highest net monetary benefit (52%) compared to open appendectomy (18%) and nonoperative management (30%). The incremental net economic benefit for laparoscopy was $93.7 per patient [23].

Sugiura et al. [26] conducted a study in Japan to assess the cost‐effectiveness of surgical treatment versus nonoperative management with and without initial laparoscopy. The cost of nonsurgical management with and without initial laparoscopy was $984 and $1219, respectively. Surgical treatment was associated with a marginal 0.005% increase in price. The study considered a WTP threshold of $100,000, with incremental cost‐effectiveness results of $172,992 and $462,843 for nonoperative management with and without initial laparoscopy, respectively. The study concluded that surgical treatment (appendectomy) was more cost‐effective than nonsurgical management with or without initial laparoscopy [26].

Ali et al. [25] conducted a CEA comparing open appendectomy to antibiotic therapy for AA in England. Their study highlighted the need for further research on the optimal treatment for AA patients. The analysis showed that antibiotic therapy instead of appendectomy saved £23,278 per patient after a 1‐year follow‐up. Sensitivity analysis, accounting for posttreatment complications, identified antibiotic treatment as the dominant strategy [26].

Scotts et al. (2019) investigated the cost‐effectiveness of different treatment approaches for AA in the American healthcare system. The study compared laparoscopy, outpatient antibiotic therapy, and inpatient antibiotic therapy using a Markov model with a lifetime horizon from the payer's perspective. The findings indicated that the lifetime cost of outpatient antibiotic therapy for AA was $233,000. In comparison, laparoscopy and nonsurgical management with hospitalization incurred additional costs of $2500 and $7300, respectively. Patients receiving outpatient antibiotic therapy had an adjusted quality of life of 24.927 years. The study concluded that laparoscopy was more cost‐effective than outpatient nonsurgical management, with an additional cost of $32,000 per QALY compared to hospitalization [18].

## 4 Discussion

The present research conducted a comprehensive review of studies focusing on the economic evaluation of treatments for AA, identifying 11 studies with a consistently high level of quality. The findings from these studies suggest that, in terms of cost‐effectiveness, laparoscopy emerges as the most economically efficient option, followed by antibiotic therapy (nonoperative management) and open appendectomy, respectively. Among the 11 studies analyzed, five studies provide compelling evidence supporting the cost‐effectiveness of laparoscopy [17, 18, 23, 24, 27], while five studies advocate for the cost‐effectiveness of nonoperative management [19, 20, 21, 22, 25], and one study supports the cost‐effectiveness of open appendectomy [26].

In the comparison between laparoscopy and open appendectomy, findings from 5 studies indicate that laparoscopy stands out as the more cost‐effective choice [17, 23, 24, 27], with only one study suggesting open appendectomy as a cost‐effective alternative [26]. These results are derived from the analysis of four studies conducted in Spain, Colombia, and the USA. Two studies evaluated the economic evaluation of these two options from the service provider's perspective. In contrast, one study examined it from the payer's viewpoint, and another considered the health system's perspective. Across these studies, which had time horizons ranging from 5 to 10 years, a consistent pattern emerged demonstrating the cost‐effectiveness of laparoscopy over appendectomy.

When comparing laparoscopy with nonsurgical management (antibiotic therapy), two studies indicate that laparoscopy is the more cost‐effective option [18, 23]. However, the evidence regarding the consistent cost‐effectiveness of laparoscopy over antibiotic therapy remains inconclusive, as other studies conducted on varied patient groups and employing diverse economic models present conflicting results on this matter [19, 20, 21].

Five studies utilized the decision tree economic model to evaluate AA treatment interventions’ short‐term and long‐term effects. Antibiotic therapy emerged as the more cost‐effective option in all five studies [19, 20, 21, 22, 24]. Additionally, three studies employed the Markov model to assess the long‐term effects of therapeutic interventions. Among these studies, two identified laparoscopy as a cost‐effective option, while one indicated open appendectomy as the more cost‐effective choice [18, 23, 26].

Six studies were conducted in the US from the perspective of the countries under investigation. Among these, two studies supported laparoscopy [17, 18], while four studies favored nonoperative management (antibiotic therapy) [19, 20, 21, 22]. Two studies were conducted in Colombia, both of which yielded results in support of laparoscopic intervention [23, 24]. Additionally, one study was conducted in Spain [27], Japan [26], and England [25]. The study from Spain reported support for laparoscopy, while the studies from Japan and England favored nonoperative management (antibiotic therapy).

The lack of high‐quality research findings and detailed information across various countries and variables in this field limits the study. While the study offers valuable insights, it is essential to recognize several constraints. Firstly, the findings are based solely on English‐language publications, potentially introducing a language bias. Moreover, only one study approached costs from a social perspective [21], focusing specifically on the age group under 18 in the USA. Additionally, the generalizability of cost‐effectiveness findings remains debatable, given that most studies included in the analysis were conducted in high‐income countries, underscoring the need for further investigations in low‐ and middle‐income nations. Variations in healthcare systems and WTP thresholds among different countries further complicate the generalizability of the results. The granularity of cost assessments varies across studies. While some provide a detailed breakdown of costs, including surgical treatment, hospitalization, and imaging, others aggregate these costs into a global estimate without itemizing individual components. This variation can affect the comparability of cost‐effectiveness findings and underscores the importance of including detailed cost components in future studies.

## 5 Conclusion

Based on the findings of this study, laparoscopic therapeutic intervention seems to be a more cost‐effective option in most cases compared to open appendectomy for the treatment of AA patients. However, it is essential to note that in specific patient groups, such as individuals under 18 years old or based on disease severity, nonoperative management (antibiotic therapy) may be a more cost‐effective choice compared to laparoscopy and open appendectomy. Overall, it is evident that further studies examining the cost‐effectiveness of AA treatment interventions are necessary, incorporating actual epidemiological data specific to the countries in question for accurate input parameters. Additionally, we suggest conducting cost‐effectiveness studies comparing laparoscopic and appendectomy approaches while considering costs and benefits from a social perspective.

## Author Contributions

**Sajad Moeini:** investigation, data curation, methodology, writing – original draft, formal analysis. **Hasan Abolghasem Gorji:** Supervision, project administration, writing – original draft. **Mohmmad Veysi Sheikhrobat:** data curation, investigation, writing – original draft. **Aziz Rezapour:** conceptualization, methodology, writing – original draft. **Aghdas Souresrafil:** data curation, methodology, formal analysis, writing – original draft. **Mohammad Barzegar:** writing – original draft, writing – review and editing.

## Ethics Statement

This study approved by the National Committee of Ethics in Biomedical Research (IR.IUMS.REC.1401.1387) and was registered in PROSPERO: International Prospective Register of Systematic Reviews and was assigned a registry ID CRD42023390140.

## Conflicts of Interest

The authors declare no conflicts of interest.

## Transparency Statement

The lead author Sajad Moeini affirms that this manuscript is an honest, accurate, and transparent account of the study being reported; that no important aspects of the study have been omitted; and that any discrepancies from the study as planned (and, if relevant, registered) have been explained.

## Supporting information

Appendix 1.

## Data Availability

The data that support the findings of this study are available on request from the corresponding author. The data are not publicly available due to privacy or ethical restrictions.
