# Supplementary material for: Economic Evaluation of Acute Appendicitis Therapeutic Interventions: A Systematic Review
Source: Health Sci Rep. 2025 May 5;8(5):e70815. doi: 10.1002/hsr2.70815 (PMC12051430; doi:10.1002/hsr2.70815)
Supplement: Supplementary file 1 — Appendix 1. [file HSR2-8-e70815-s001.docx]

Appendix 1: Search strategy of systematic review databases of economic evaluation studies of acute appendicitis

| Database | Search strategy |
| --- | --- |
| PubMed | (Cost[tiab] OR “Cost analysis”[tiab] OR “cost comparison”[tiab] OR (Cost[tiab] AND analysis[tiab] ) OR cost-effectiveness[tiab] OR "cost effectiveness"[tiab] OR cost-utility[tiab] OR "cost utility"[tiab] OR cost-benefit[tiab] OR "cost benefit"[tiab] OR "economic evaluation"[tiab] OR "health resource allocation"[tiab] OR "health economic"[tiab] OR (economic[tiab] AND medical[tiab]) OR economic*[ti] OR "decision analysis"[tiab] OR decision-analytic[tiab] OR “health technology assessment”[tiab]) AND (laparoscopy[tiab] OR “laparoscopic appendectomy”[tiab] OR “Laparoscopic”[tiab] OR Laparoscopies[tiab] OR Celioscopy[tiab] OR Celioscopies[tiab] OR Peritoneoscopy[tiab] OR Peritoneoscopies[tiab] OR (“Surgical Procedures”[tiab] AND Laparoscopic[tiab]) OR “Laparoscopic Surgical Procedure”[tiab] OR (Procedure[tiab] AND “Laparoscopic Surgical”[tiab]) OR (Procedures[tiab] AND “Laparoscopic Surgical”[tiab]) OR (Surgery[tiab] AND Laparoscopic[tiab]) OR “Laparoscopic Surgical Procedures”[tiab] OR “Laparoscopic Surgery”[tiab] OR “Laparoscopic Surgeries”[tiab] OR (Surgeries[tiab] AND Laparoscopic[tiab]) OR “Laparoscopic Assisted Surgery”[tiab] OR “Laparoscopic Assisted Surgeries”[tiab] OR (Surgeries[tiab] AND “Laparoscopic Assisted”[tiab]) OR (Surgery[tiab] AND “Laparoscopic Assisted”[tiab]) OR (“Surgical Procedure”[tiab] AND Laparoscopic[tiab]) OR “minimally invasive”[tiab] OR “keyhole surgery”[tiab] OR “nonoperative management”[tiab] OR Antibiotic*[tiab] OR “intravenous fluids”[tiab] OR “bowel rest”[tiab] OR nonoperative[tiab] OR nonsurgical[tiab] OR conservative[tiab]) AND (Appendicitis[tiab] OR “Ruptured Appendicitis”[tiab] OR (Appendicitis[tiab] AND Ruptured[tiab]) OR “Perforated Appendicitis”[tiab] OR (Appendicitis[tiab] AND Perforated[tiab]) OR “complicated appendicitis”[tiab] OR “gangrenous appendicitis”[tiab] OR “Uncomplicated Appendicitis”[tiab] OR Nonperforated[tiab]) AND 1990/01/01:2023/01/01[dp] |
| Web of Science | (TS=(Cost) OR TS=(“Cost analysis”) OR TS=(“cost comparison”) OR (TS=(Cost) AND TS=(analysis)) OR TS=(cost-effectiveness) OR TS=("cost effectiveness") OR TS=(cost-utility) OR TS=("cost utility") OR TS=(cost-benefit) OR TS=("cost benefit") OR TS=("economic evaluation") OR TS=("health resource allocation") OR TS=("health economic") OR (TS=(economic) AND TS=(medical)) OR TI=(economic*) OR TS=("decision analysis") OR TS=(decision-analytic) OR TS=(“health technology assessment”)) AND (TS=(laparoscopy) OR TS=(“laparoscopic appendectomy”) OR TS=( “Laparoscopic”) OR TS=(Laparoscopies) OR TS=(Celioscopy) OR TS=(Celioscopies) OR TS=(Peritoneoscopy) OR TS=(Peritoneoscopies) OR (TS=(“Surgical Procedures”) AND TS=(Laparoscopic)) OR TS=( “Laparoscopic Surgical Procedure”) OR (TS=(Procedure) AND TS=(“Laparoscopic Surgical”)) OR (TS=(Procedures) AND TS=( “Laparoscopic Surgical”)) OR (TS=(Surgery) AND TS=(Laparoscopic)) OR TS=(“Laparoscopic Surgical Procedures”) OR TS=(“Laparoscopic Surgery”) OR TS=(“Laparoscopic Surgeries”) OR (TS=(Surgeries) AND TS=(Laparoscopic)) OR TS=( “Laparoscopic Assisted Surgery”) OR TS=(“Laparoscopic Assisted Surgeries”) OR (TS=(Surgeries) AND TS=(“Laparoscopic Assisted”)) OR (TS=(Surgery) AND TS=( “Laparoscopic Assisted”)) OR (TS=(“Surgical Procedure”) AND TS=(Laparoscopic)) OR TS=( “minimally invasive”) OR TS=(“keyhole surgery”) OR TS=(“nonoperative management”) OR TS=(Antibiotic*) OR TS=(“intravenous fluids”) OR TS=(“bowel rest”) OR TS=(nonoperative) OR TS=(nonsurgical) OR TS=(conservative)) AND (TS=(Appendicitis) OR TS=(“Ruptured Appendicitis”) OR (TS=(Appendicitis) AND TS=(Ruptured)) OR TS=(“Perforated Appendicitis”) OR (TS=(Appendicitis) AND TS=(Perforated)) OR TS=(“complicated appendicitis”) OR TS=(“gangrenous appendicitis”) OR TS=(“Uncomplicated Appendicitis”) OR TS=(Nonperforated)) AND PY=(1990-2023) |
| Scopus | (TITLE-ABS(Cost) OR TITLE-ABS(“Cost analysis”) OR TITLE-ABS(“cost comparison”) OR (TITLE-ABS(Cost) AND TITLE-ABS(analysis)) OR TITLE-ABS(cost-effectiveness) OR TITLE-ABS("cost effectiveness") OR TITLE-ABS(cost-utility) OR TITLE-ABS("cost utility") OR TITLE-ABS(cost-benefit) OR TITLE-ABS("cost benefit") OR TITLE-ABS("economic evaluation") OR TITLE-ABS("health resource allocation") OR TITLE-ABS("health economic") OR (TITLE-ABS(economic) AND TITLE-ABS(medical)) OR TITLE(economic*) OR TITLE-ABS("decision analysis") OR TITLE-ABS(decision-analytic) OR TITLE-ABS(“health technology assessment”)) AND (TITLE-ABS(laparoscopy) OR TITLE-ABS(“laparoscopic appendectomy”) OR TITLE-ABS( “Laparoscopic”) OR TITLE-ABS(Laparoscopies) OR TITLE-ABS(Celioscopy) OR TITLE-ABS(Celioscopies) OR TITLE-ABS(Peritoneoscopy) OR TITLE-ABS(Peritoneoscopies) OR (TITLE-ABS(“Surgical Procedures”) AND TITLE-ABS(Laparoscopic)) OR TITLE-ABS( “Laparoscopic Surgical Procedure”) OR (TITLE-ABS(Procedure) AND TITLE-ABS(“Laparoscopic Surgical”)) OR (TITLE-ABS(Procedures) AND TITLE-ABS( “Laparoscopic Surgical”)) OR (TITLE-ABS(Surgery) AND TITLE-ABS(Laparoscopic)) OR TITLE-ABS(“Laparoscopic Surgical Procedures”) OR TITLE-ABS(“Laparoscopic Surgery”) OR TITLE-ABS(“Laparoscopic Surgeries”) OR (TITLE-ABS(Surgeries) AND TITLE-ABS(Laparoscopic)) OR TITLE-ABS( “Laparoscopic Assisted Surgery”) OR TITLE-ABS(“Laparoscopic Assisted Surgeries”) OR (TITLE-ABS(Surgeries) AND TITLE-ABS(“Laparoscopic Assisted”)) OR (TITLE-ABS(Surgery) AND TITLE-ABS( “Laparoscopic Assisted”)) OR (TITLE-ABS(“Surgical Procedure”) AND TITLE-ABS(Laparoscopic)) OR TITLE-ABS( “minimally invasive”) OR TITLE-ABS(“keyhole surgery”) OR TITLE-ABS(“nonoperative management”) OR TITLE-ABS(Antibiotic*) OR TITLE-ABS(“intravenous fluids”) OR TITLE-ABS(“bowel rest”) OR TITLE-ABS(nonoperative) OR TITLE-ABS(nonsurgical) OR TITLE-ABS(conservative)) AND (TITLE-ABS(Appendicitis) OR TITLE-ABS(“Ruptured Appendicitis”) OR (TITLE-ABS(Appendicitis) AND TITLE-ABS(Ruptured)) OR TITLE-ABS(“Perforated Appendicitis”) OR (TITLE-ABS(Appendicitis) AND TITLE-ABS(Perforated)) OR TITLE-ABS(“complicated appendicitis”) OR TITLE-ABS(“gangrenous appendicitis”) OR TITLE-ABS(“Uncomplicated Appendicitis”) OR TITLE-ABS(Nonperforated)) AND (PUBYEAR > 1989 AND PUBYEAR < 2024) |
| Embase | (Cost:ti,ab OR “Cost analysis”:ti,ab OR “cost comparison”:ti,ab OR (Cost:ti,ab AND analysis:ti,ab ) OR cost-effectiveness:ti,ab OR "cost effectiveness":ti,ab OR cost-utility:ti,ab OR "cost utility":ti,ab OR cost-benefit:ti,ab OR "cost benefit":ti,ab OR "economic evaluation":ti,ab OR "health resource allocation":ti,ab OR "health economic":ti,ab OR (economic:ti,ab AND medical:ti,ab) OR economic*:ti OR "decision analysis":ti,ab OR decision-analytic:ti,ab OR “health technology assessment”:ti,ab) AND (laparoscopy:ti,ab OR “laparoscopic appendectomy”:ti,ab OR “Laparoscopic”:ti,ab OR Laparoscopies:ti,ab OR Celioscopy:ti,ab OR Celioscopies:ti,ab OR Peritoneoscopy:ti,ab OR Peritoneoscopies:ti,ab OR (“Surgical Procedures”:ti,ab AND Laparoscopic:ti,ab) OR “Laparoscopic Surgical Procedure”:ti,ab OR (Procedure:ti,ab AND “Laparoscopic Surgical”:ti,ab) OR (Procedures:ti,ab AND “Laparoscopic Surgical”:ti,ab) OR (Surgery:ti,ab AND Laparoscopic:ti,ab) OR “Laparoscopic Surgical Procedures”:ti,ab OR “Laparoscopic Surgery”:ti,ab OR “Laparoscopic Surgeries”:ti,ab OR (Surgeries:ti,ab AND Laparoscopic:ti,ab) OR “Laparoscopic Assisted Surgery”:ti,ab OR “Laparoscopic Assisted Surgeries”:ti,ab OR (Surgeries:ti,ab AND “Laparoscopic Assisted”:ti,ab) OR (Surgery:ti,ab AND “Laparoscopic Assisted”:ti,ab) OR (“Surgical Procedure”:ti,ab AND Laparoscopic:ti,ab) OR “minimally invasive”:ti,ab OR “keyhole surgery”:ti,ab OR “nonoperative management”:ti,ab OR Antibiotic*:ti,ab OR “intravenous fluids”:ti,ab OR “bowel rest”:ti,ab OR nonoperative:ti,ab OR nonsurgical:ti,ab OR conservative:ti,ab) AND (Appendicitis:ti,ab OR “Ruptured Appendicitis”:ti,ab OR (Appendicitis:ti,ab AND Ruptured:ti,ab) OR “Perforated Appendicitis”:ti,ab OR (Appendicitis:ti,ab AND Perforated:ti,ab) OR “complicated appendicitis”:ti,ab OR “gangrenous appendicitis”:ti,ab OR “Uncomplicated Appendicitis”:ti,ab OR Nonperforated:ti,ab) AND [1990-2023]/PY |
